# Taxonomic and phenotypic analysis of bifidobacteria isolated from IBD patients as potential probiotic strains

**DOI:** 10.1186/s12866-024-03368-4

**Published:** 2024-06-29

**Authors:** Sabine Bosselaar, Lucile Dhelin, Ellena Dautel, Marie Titecat, Stéphanie Duthoy, Marie Stelmaszczyk, Nathan Delory, Madeleine De Sousa Violante, François Machuron, Hassina Ait-Abderrahim, Pierre Desreumaux, Benoit Foligné, Céline Monnet

**Affiliations:** 1 Lesaffre International - Lesaffre Institute of Science and Technology, 101 Rue de Menin, 59706 Marcq-en-Barœul, France; 2 Univ. Lille, Inserm, CHU Lille, U1286 – INFINITE - Institute for Translational Research in Inflammation, 59000 Lille, France; 3 Department of Hepato-Gastroenterology, Lille University Hospital, 59037 Lille, France

**Keywords:** Bifidobacteria, Inflammatory Bowel Diseases, Microbiota, Culturomics, Functional characterization

## Abstract

**Background:**

Inflammatory Bowel Diseases (IBD) are a major public health issue with unclear aetiology. Changes in the composition and functionality of the intestinal microbiota are associated with these pathologies, including the depletion of strict anaerobes such as *Feacalibacterium prausnitzii*. Less evidence is observed for depletion in other anaerobes, among which bifidobacteria. This study characterized the taxonomic and functional diversity of bifidobacteria isolated from the human intestinal microbiota in active and non-active IBD patients by a culturomics approach and evaluated if these bifidobacteria might be used as probiotics for gut health.

**Results:**

A total of 341 bifidobacteria were isolated from the intestinal microbiota of IBD patients (52 Crohn’s disease and 26 ulcerative colitis patients), with a high proportion of *Bifidobacterium dentium* strains (28% of isolated bifidobacteria). In ulcerative colitis, the major species identified was *B. dentium* (39% of isolated bifidobacteria), in active and non-active ulcerative colitis. In Crohn’s disease, *B. adolescentis* was the major species isolated from non-active patients (40%), while similar amounts of *B. dentium* and *B. adolescentis* were found in active Crohn’s disease patients. The relative abundance of *B. dentium* was increased with age, both in Crohn’s disease and ulcerative colitis and active and non-active IBD patients. Antibacterial capacities of bifidobacteria isolated from non-active ulcerative colitis against *Escherichia coli* LF82 and *Salmonella enterica* ATCC 14028 were observed more often compared to strains isolated from active ulcerative colitis. Finally, *B. longum* were retained as strains with the highest probiotic potential as they were the major strains presenting exopolysaccharide synthesis, antibacterial activity, and anti-inflammatory capacities. Antimicrobial activity and EPS synthesis were further correlated to the presence of antimicrobial and EPS gene clusters by in silico analysis.

**Conclusions:**

Different bifidobacterial taxonomic profiles were identified in the microbiota of IBD patients. The most abundant species were *B. dentium*, mainly associated to the microbiota of ulcerative colitis patients and *B. adolescentis*, in the intestinal microbiota of Crohn’s disease patients. Additionally, the relative abundance of *B. dentium* significantly increased with age. Furthermore, this study evidenced that bifidobacteria with probiotic potential (antipathogenic activity, exopolysaccharide production and anti-inflammatory activity), especially *B. longum* strains, can be isolated from the intestinal microbiota of both active and non-active Crohn’s disease and ulcerative colitis patients.

**Supplementary Information:**

The online version contains supplementary material available at 10.1186/s12866-024-03368-4.

## Background

Inflammatory Bowel Diseases (IBD), including Crohn’s disease (CD) and ulcerative colitis (UC) are multifactorial disabling diseases representing a major public health issue. Both diseases represent a chronic relapsing–remitting condition affecting the gut. Scoring systems based on clinical, biological, endoscopic, radiologic and histologic observations have been developed and validated to describe disease activity; however, the definitions of relapse and remission remain still under debate [1]. IBD have unclear aetiology implicating genetic susceptibility, environmental factors, and influence of gut microbiota, contributing to a chronic inflammation of the gastrointestinal tract [2–4]. Hence, the intestinal microbiota has gained much attention as an important factor in disease development, but also as a focus for new emerging therapeutic approaches with the development of probiotics, prebiotics or synbiotics.

Dysbiosis observed in the intestinal microbiota of IBD patients may play an important role in IBD pathogenesis. The intestinal microbiota of IBD patients have shown decreases in *Firmicutes* such as *Faecalibacterium prausnitzii*, *Blautia faecis* and *Roseburia inulinivorans*, increases in *Proteobacteria*, mainly enterobacteria such as *Escherichia coli* (with an increase in pathobiontic Adherent Invasive *E. coli*), and also *Fusobacterium* spp*., Ruminococcus gnavus* or pathogens such as *Clostridium difficile*, *Campylobacter* or *Salmonella*. This dysbiosis leads to functional differences as bacteria with beneficial activities such as short chain fatty acid production and anti-inflammatory capacities are decreased while bacteria with opportunistic detrimental functions such as pro-inflammatory capacities or mucolytic activity are increased [5–7].

Generally, the intestinal microbiota of IBD patients are enriched in aerobic or aerotolerant bacteria and are depleted in strict anaerobes and it has been largely shown that *F. prausnitzii* is reduced in the intestinal microbiota of patients with IBD[5, 6]. Nevertheless, less evidence is made for other beneficial anaerobic bacteria, such as bifidobacteria. Several studies have shown decreased levels in bifidobacteria in IBD [8], while other increased abundance of bifidobacteria in UC (but not CD) compared to healthy controls [9–11]. To our knowledge, only very limited data was published focusing specifically on bifidobacteria analysis within the microbiota of IBD patients at species level. Gueimonde et al*.* characterized intestinal adherent strains in colon cancer, IBD and diverticulitis patients and detected bifidobacteria in all IBD samples with a majority of *B. longum* and *B. bifidum* [12]. Furthermore, Duranti et al*.* identified a reduction in *B. bifidum* in the intestinal microbiota of ulcerative colitis [13]. More studies investigated the probiotic effects of bifidobacteria in IBD. In different animal colitis models, several bifidobacteria strains reduced symptoms, improved histological colon observations and reduced the pro-inflammatory response (reviewed in [14, 15]). Human clinical trials also evidenced beneficial roles of supplementation of *B. longum* and *B. breve* strains in UC [16–19] and CD [20], with decreased disease activity, decreased endoscopic inflammation and anti-inflammatory effects. While bifidobacteria are known as one of the major known beneficial bacteria in the human intestinal microbiota and are largely studied for their probiotic application in IBD, the natural occurrence of bifidobacteria in IBD microbiota has not been fully characterized.

The aim of this in-depth bifidobacteria characterization in IBD patients by a culturomics approach was two-fold: evaluate the taxonomic and functional diversity of bifidobacteria in active and non-active IBD patients and reveal if these bifidobacteria could potentially be used as probiotics in gut health. This work presents a culturomics approach to characterize the bifidobacteria microbiota of IBD patients in faecal samples collected during medical care at Lille University Hospital. Most studies profiling dysbiosis in IBD were conducted by metagenomic approaches. These techniques give a good overall insight into microbiota composition but lack specificity for characterization of minor genera at species level and present many biases depending on method of analysis [21, 22]. Here, a culturomics approach allows us to focus on the bifidobacterial subpopulation of the intestinal microbiota, to characterize their functional potential in vitro and identify potential probiotic strains. We focused on three beneficial health effects known in bifidobacteria for functional characterization: exopolysaccharide (EPS) production, anti-pathogenic capacities, and immunomodulatory effects. Indeed, bifidobacteria produce a wide range of exopolysaccharides, mainly composed of galactose, glucose and rhamnose, with shown beneficial health effects such as protective effects favouring persistence in the gut, modulation of the microbiota, anti-pathogenic activity and immunomodulatory effects [23, 24]. We evaluated anti-pathogenic activity of bifidobacteria isolated from IBD patients on a IBD associated pathobiont, *E. coli* LF82 [25–27], but also on one of the major foodborne pathogens, *Salmonella enterica* [28, 29]. Anti-inflammatory potential was evaluated by measuring decreased synthesis of IL-8 (a pro-inflammatory chemokine activating neutrophils) in intestinal epithelial HT-29 cells, an extensively recognised screening model for anti-inflammatory probiotics [30–33]. A reduction in IL-8 synthesis is of interest in IBD patients as IL-8 increase has been shown in active UC and CD [34, 35] but also in global gut health, as IL-8 secretion is also increased in enteropathogen-induced inflammation [36, 37]. We also established bacterial fitness and oxygen tolerance to identify if bacteria isolated from intestinal inflamed environments could have adapted to their environment and present high tolerance levels and fitness and thus be particularly suitable as future probiotic candidates regarding industrial processes.

## Methods

### IBD patients

This study included patients with medical follow-up by a gastroenterologist at the Lille University Hospital (France) between the 2nd February 2021 and the 28th June 2022. Patients diagnosed with CD or UC, without antibiotic treatment in the last month, that provided faeces for medical care were included in this study. Faeces were collected for research purpose under secondary use with informed consent of the patients. For all samples, a database containing the following metadata was created: Pathology, age, sex, duration of pathology, location of inflammation, symptomatic observations, and fecal calprotectin quantification. Patients were considered in active CD or UC if fecal calprotectin quantification was ≥ 250 µg/g (with or without clinical symptoms) and in non-active CD or UC when fecal calprotectin quantification was < 250 µg/g and patients did not present symptomatic clinical gastrointestinal disorders. One patient with low fecal calprotectin quantification (< 250 µg/g) but presenting gastrointestinal disorders was excluded from analysis.

Analysis was performed on a total of 138 samples collected from 103 IBD patients: 98 samples were collected from CD (66% women, age 43.5 ± 13.6) and 40 from UC (65% women, age 49.6 ± 16.7), from patients with non-active (CD = 59, UC = 22) and active disease (CD = 39, UC = 18). Diversity of patients per pathology is described in Table 1.

**Table 1:** Characteristics of sample origin (*N* = 138 fecal samples) from IBD patients

	**Crohn's Disease**			**Ulcerative Colitis**		
	Non active	Active	All CD patients	Non active	Active	All UC patients
**Number of samples**	**59 (60%)**	**39 (40%)**	**98 (100%)**	**22 (55%)**	**18 (45%)**	**40 (100%)**
**Sex**						
Men	22 (37%)	11 (28%)	33 (34%)	10 (45%)	4 (22%)	14 (35%)
Women	37 (63%)	28 (72%)	65 (66%)	12 (55%)	14 (78%)	26 (65%)
**Age**						
< 50 years	38 (64%)	20 (51%)	58 (59%)	10 (45%)	13 (72%)	23 (58%)
≥ 50 years	21 (36%)	19 (49%)	40 (41%)	12 (55%)	5 (28%)	17 (42%)
**Gastro-intestinal disorders**						
Yes		16 (41%)	16 (16%)		9 (50%)	9 (23%)
No	59 (100%)	22 (56%)	81 (83%)	22 (100%)	8 (44%)	30 (75%)
Unknown		1 (3%)	1 (1%)		1 (6%)	1 (2%)
**Location of inflammation**						
Small intestine only	40 (68%)	19 (49%)	59 (60%)			
Colon	19 (32%)	20 (51%)	39 (40%)	22 (100%)	18 (100%)	40 (100%)
**Duration of pathology**						
< 5 years	6 (10%)	7 (18%)	13 (13%)	2 (9%)	2 (11%)	4 (10%)
5–15 years	27 (46%)	11 (28%)	38 (39%)	11 (50%)	10 (56%)	21 (53%)
15–25 years	14 (24%)	12 (31%)	26 (27%)	5 (23%)	3 (17%)	8 (20%)
≥ 25 years	11 (19%)	3 (8%)	14 (14%)	4 (18%)	2 (11%)	6 (15%)
Unknown	1 (2%)	6 (15%)	7 (7%)		1 (6%)	1 (2%)
**Strain isolation**						
Samples with bifidobacteria isolated	29 (49%)	23 (59%)	52 (53%)	14 (64%)	12 (67%)	26 (65%)
Samples without bifidobacteria isolated	30 (51%)	16 (41%)	46 (47%)	8 (36%)	6 (33%)	14 (35%)
** Total number of strains isolated**	**379 (63%)**	**219 (37%)**	**598 (100%)**	**122 (50%)**	**121 (50%)**	**243 (100%)**
**Strain isolation**						
Total number of bifidobacteria isolated	131 (35%)	104 (47%)	235 (39%)	48 (39%)	58 (48%)	106 (44%)

### Bacteria enumeration and strain isolation of fecal samples

Fecal samples were treated within 5h after collection. One g of fecal material was homogenized in 9mL of freshly regenerated cysteine diluent (heated at 90°C for 20 min and cooled down right before use). Serial dilutions were plated on freshly prepared Columbia Beerens medium [38], for bifidobacteria enriched enumeration and Columbia Blood Agar for total anaerobic bacteria enumeration*.* Plates were incubated at 37°C for 6 days in anaerobic jars. From Columbia Beerens agar plates, up to 10 colonies were randomly selected per fecal sample, on plates presenting isolated colonies. When different morphologies were picked, number of colonies picked per morphology were proportional to the representativity of this morphology in the sample to minimize biases in diversity ratios. Strains were then subcultured 3 times on agar plates from an isolated colony to guarantee purity on De Man-Ragosa-Sharpe (MRS) medium (Biomérieux, France) supplemented with 0.05% L-cysteine HCl at pH 6.5 (MRSc) in anaerobic conditions (Don Withley Anaerobic Workstation, 37°C, 10% H_2_, 10% CO_2_, 80% N_2_).

### Strain identification

Strains were taxonomically identified by Matrix Assisted Laser Desorption Ionization—Time of Flight (MALDI-TOF) [39]. Bacterial isolates were cultivated on MRSc agar plates. Protein extraction procedure was performed on each isolate. 1 µL of the extract were deposited on a 96-well target plate and analyzed with the MALDI-TOF mass spectrometer according to the manufacturer’s recommendations (Brüker, Wissembourg, France). Species were identified using an accurate identification score > 1.9.

Identification was confirmed by Whole Genome Sequencing. Total genomic DNA was extracted from pure bacterial cultures using EZ-96 isolation kit tissue DNA from Omega Biotek following provider instructions with exception of the lysis step. Lysis was achieved by enzymatic reaction (20 mM Tris, 2 mM EDTA, pH 8 + 20 mg/mL lysozyme + 1,2% Triton, incubation 30 min at 30 °C) prior to starting the provider process. Sequencing libraries were prepared using Illumina DNA Prep kit following providers instructions. Isolated DNA and libraries samples were quantified and validated using the Qubit fluorometric method (Invitrogen). Sequencing of an equimolar pool of multiplexed samples was realized on a Nextseq 550 lllumina sequencer in paired-end sequencing mode producing reads of 149 bases using PhiX as control. Illumina short reads were de novo assembled using Spades (v3.15), and quality controls were performed on all genomes with Quast (v5.1). Species were identified using BLAST on the whole genome GTDB database and assessed using the ANI (Average Nucleotide Identity) metric calculated by fastANI (> 96% to type strain) [40]. To check species identification, a phylogenetic tree was created by unweighted paired group mean arithmetic (upgma) method using MASH distances and represented using ggtree.

### In vitro functional characterization

For in vitro functional characterization, bifidobacteria were routinely grown in liquid MRSc, autoclaved and stored under N_2_ atmosphere. Bifidobacteria were inoculated at 10% in a 96-well DeepWell, precultured for 24h, Optical Density at 620nm (OD_600nm_) adjusted at 0.08 and cultured for another 18h to reach the end of exponential growth phase. Bifidobacteria were routinely incubated at 37°C in anaerobic conditions (10% H_2_, 10% CO_2_, 80% N_2_, Bact-R Plus System).

### Growth characterization and oxygen tolerance

To determine growth curves, OD_600nm_ adjusted precultures were incubated in anaerobic conditions (10% H_2_, 10% CO_2_, 80% N_2_, Don Withley Anaerobic Workstation) and OD_620nm_ was measured every 20min (Byonoy Absorbance 96 plate reader). Growth parameters were calculated with the Growthcurver R package [41].

To determine oxygen tolerance, OD_600nm_ adjusted precultures were incubated in microaerophilic conditions (5% of residual O_2_, Bact-R Plus Systems) and OD_600nm_ was read after 24h and 48h of incubation at 37°C. To evaluate oxygen tolerance, ratio of OD_600nm_ in microaerophilic conditions / OD_620nm_ in anaerobic conditions was calculated for each strain. Strains with ratios > 0.6 were considered tolerant to growth in microaerophilic conditions and strains with ratios < 0.2 were considered intolerant to growth in microaerophilic conditions.

### Exopolysaccharides production

EPS producing strains were identified by their mucoid phenotype when grown on agar plates as described by Ruas Madiedo [42]. Briefly, bifidobacteria precultures were spotted on 0.05% L-cysteine HCl—MRS agar plates with 20g/L glucose, 60g/L glucose, 100g/L sucrose, 100g/L maltose or 100g/L lactose. Strains presenting a mucoid phenotype after 72h incubation (37°C, 10% H_2_, 10% CO_2_, 80% N_2_, Bact-R Plus System) were considered EPS producing strains. Strains forming white dry colonies were considered non-EPS producing strains. EPS phenotype of strains presenting an intermediate phenotype (“classic” white colonies) were not established.

In silico, exopolysaccharide gene clusters were predicted for strains presenting an EPS producing phenotype in vitro. Priming glycosyltransferase encoding genes were searched by BLAST against the B624_0342 gene from *B. longum* 35624 (accession number CP013673). Extended genomic regions around the priming glycosyltransferase gene were manually analyzed to identify potential EPS gene cluster regions. Genetic regions were annotated by combining genomic annotations from Prokka [43]. BLAST against amino acid sequences of EPS related genes described in other bifidobacterial gene clusters [23, 44, 45] and InterPro [46].

### Antibacterial activity

To test the antibacterial activity, co-culture spot plate assays were set up, similarly to described by O’Riordan and Fitzgerald [47]. *Bifidobacterium* precultures were spotted on MRS (Biomérieux, France) agar plates supplemented with 0.05% L-cysteine HCl pH 6.5 and grown for 24h at 37°C in anaerobic conditions (37°C, 10% H_2_, 10% CO_2_, 80% N_2_, Bact-R Plus System) before addition of an overlay of *Salmonella enterica subsp. enteriditis* ATCC 14028 (inoculation at OD_600nm_ 0.005) or *E. coli* LF82 (inoculation at OD_600nm_ 0.05) in soft agar (8g/L agar) TSB or LB medium respectively. After a supplementary 24h incubation (37°C, 37°C, 10% H_2_, 10% CO_2_, 80% N_2_, Bact-R Plus System), inhibition scores were attributed according to size of inhibition zones (from 0.25 for nearly visible inhibition zones to 1 for control strain *B. longum* DSM 20219). Mean inhibition scores of replicates were calculated, and inhibition was considered strong for average scores above 0.65 and absent for scores below 0.35.

In silico, antimicrobial gene clusters were predicted using BAGEL4 (v4.0) for bacteriocins and ribosomally synthesized and post-translationally modified peptides and antiSMASH (v6) for other secondary metabolite [48, 49].

### Immunomodulatory effect

Immunomodulatory effect was evaluated on a subset of 130 strains. For this selection, groups of genetically identical strains (fastANI > 99.99%) were formed and one representative per group was included. Immunomodulatory effect was evaluated by measuring reduction of LPS-stimulated IL-8 secretion by bifidobacteria strains in a HT-29 cell culture model. This model was largely described in literature [33, 50–58] with different stimulants at various concentrations to induce inflammatory response and different incubation times. Here, the model was adapted for high throughput screening to a 96-well MTP assay and optimal amount of HT29 cells per well, cell/bacteria ratio, LPS concentration and incubation times were defined after multiple tests during method set-up. HT-29 cells (HTB-38, ATCC) were routinely cultivated in DMEM 4,5g/L D-glucose, pyruvate, GlutaMAX, 10% FCS (Gibco) at 37°C, 5% CO_2_. Bifidobacteria cultures were adjusted to OD_600nm_ = 0.25 (approximately 4.10^8^ CFU/mL) and diluted 20-fold in DMEM medium. Bacterial cell suspensions were added on previously plated and adhered HT-29 cells (40 000 cells / well in 96 well plates) at MOI 50 and incubated for 4h (37°C, 5% CO_2_). After 4h incubation, inflammation was induced by addition of 10ng/mL of lipopolysaccharide (LPS) 0111:B4 (L4391, Sigma-Aldrich). Supernatants were collected after 24h incubation (37°C, 5% CO2), filter sterilized (0.2µm) and stored at -80°C until analysis. IL-8 secretion by HT-29 cells was measured by ELISA (DuoSet ELISA Development System Human IL-8/CXCL8, DY208—R&D Systems) according to the manufacturer’s protocol. For inter-experiment normalization, results were expressed as diminution of IL-8 secretion by bifidobacteria compared to control condition (LPS-stimulated HT-29 cells without probiotic treatment).

### Statistical analysis

The number of isolated strains summarized by families were presented using percentages to consider the unbalanced numbers of patients in groups. Percentages were compared between groups using Chi^2^ tests. In case of overall significant *p*-value, post-hoc Chi^2^ tests were performed for each family with adjustment of *p*-values with Bonferroni method to control type-1 error inflation due to multiplicity testing. For functional characterization, all experiments were performed in technical and biological duplicates and mean results on the 4 values were considered for analysis.

## Results

### Bifidobacteria isolation in IBD patients

#### Similar fecal sample enumerations between active and non-active CD and UC

**Fig. 1:**
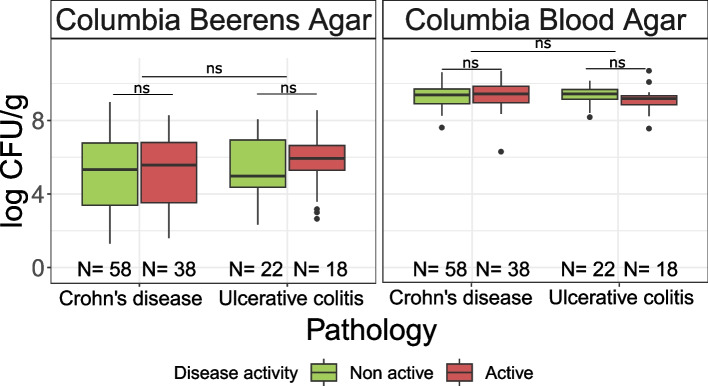
Bacterial enumeration of fecal samples on Columbia Beerens medium (Bifidobacteriaceae enrichment) and Columbia blood agar (Total bacteria) grouped by pathology and disease activity. Boxplots represent median (solid line), first and third quartile (lower and upper boundaries), lower and upper limits (whiskers, defined by 1.5* inter quartile range) and outliers (dotpoints). Wilcoxon test *p*-values: *: 0.01–0.05; **: 0.001–0.01; ***: < 0.001; ns: > 0.05. N: number of samples per group

Bacteria of fecal samples were enumerated on Columbia Beerens (*Bifidobacteriaceae* enrichment) and Columbia blood agar (Total anaerobic flora). No significant differences were observed in bacterial enumerations between samples from patients in CD and UC (Wilcoxon test *p*-value = 0.38 for Columbia Beerens Agar and 0.43 for Columbia Blood Agar). In CD and UC patient groups, no significant differences were observed in bacterial enumerations (*Bifidobacteriaceae* enrichment or total bacteria) between active or non-active patients (Wilcoxon test *p*-values > 0.05, Fig. 1).

### Bifidobacteria isolated from IBD were enriched in *B. dentium* and *B. adolescentis*

**Fig. 2:**
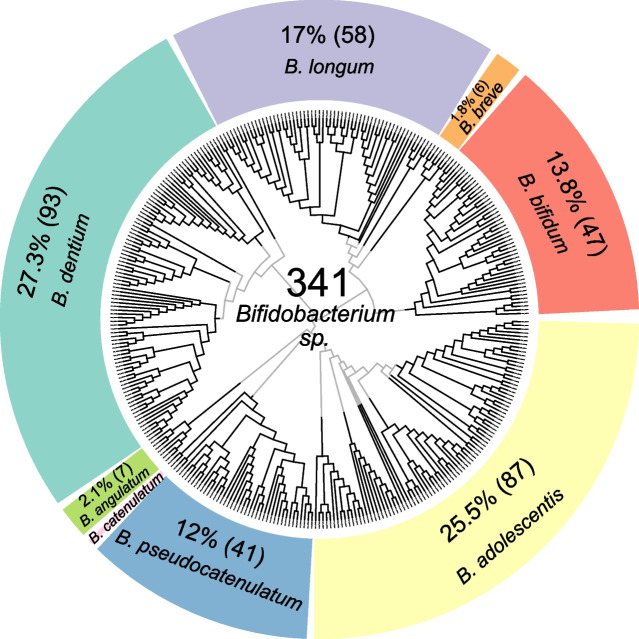
Taxonomic species identification of the 341 bifidobacteria strains isolated from 78 IBD patients (*N* = 52 Crohn’s disease patients + *N* = 26 Ulcerative colitis patients). Ratios are presented as relative abundance of bifidobacteria isolated (absolute number of bifidobacteria strains isolated)

A total of 841 strains (from 136 fecal samples) were isolated on Columbia Beerens medium, among which 341 were identified as bifidobacteria. For 58 fecal samples, only lactic acid bacteria were isolated. From the other 78 fecal samples (52 CD and 26 UC), 341 bifidobacteria were isolated. Bifidobacteria were isolated from UC and CD patients in similar ratios, with respectively 39 and 44% of total isolated strains in these patient groups identified as bifidobacteria. Fecal microbiota of IBD patients were enriched in *B. dentium* (27.3% of all bifidobacteria isolated, *n* = 93 strains), *B. adolescentis* (25.5%, *n* = 87 strains) and *B. longum* (17%, *n* = 58 strains). Other species isolated were *B. bifidum* (14%), *B. pseudocatenulatum* (12%), *B. breve* (2%), *B. angulatum* (2%) and *B. catenulatum* (1%) (Fig. 2).

### Bifidobacteria profiles differ between CD and UC and vary between remission and relapse

**Fig. 3:**
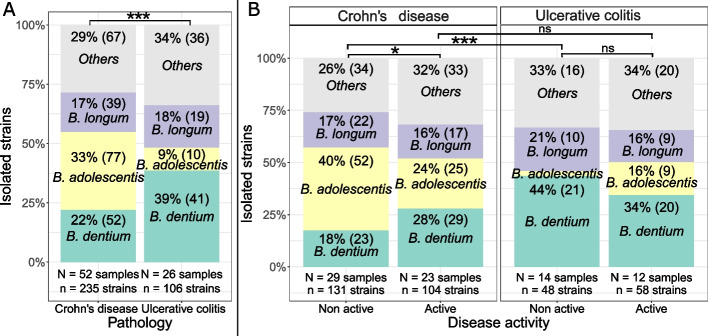
Characterization of the bifidobacteria profile in intestinal microbiota of IBD patients by (**A**) pathology or (**B**) disease activity. Ratios are presented as relative proportion of bifidobacteria isolated (absolute number of strains isolated). Ratios were not displayed for species with relative abundance ≤ 5%. Chi^2^ test *p*-values: *: 0.01–0.05; **: 0.001–0.01; ***: < 0.001; ns: > 0.05. N: number of samples from which strains were isolated. n: number of strains isolated

Bifidobacteria profiles differed between CD and UC patients (Chi^2^ test *p*-value < 0.001) due to different ratios of *B. adolescentis* (adjusted *p*-value < 0.001) and *B. dentium* (adjusted *p*-value < 0.05). In CD, a majority of *B. adolescentis* strains were isolated (33%) while in UC, a majority of *B. dentium* were isolated (39%) (Fig. 3A).

We further analyzed if these differences in bifidobacteria profiles could be due to the location of the inflammation in the digestive tract. No significant differences were identified in bifidobacteria profiles between CD patients where inflammation was located only in the small intestine or also affected the colon (Chi^2^ test *p*-value = 0.1, adjusted *p*-values per species = 1). Different bifidobacteria profiles were observed between UC patients and colic forms of CD (Chi^2^ test *p*-value < 0.001) with increased *B. adolescentis* ratios in UC (adjusted *p*-value < 0.001). In UC, most strains isolated were *B. dentium* (39%) and only 10% were *B.* *adolescentis*. In contrast, in colic forms of CD, the majority of strains isolated were identified as *B. adolescentis* strains (34%) and only 24% as *B. dentium* (data not shown). These observations were identical to global observations between UC and CD (Fig. 3A).

Bifidobacteria taxonomic profiles differed between active and non-active CD (Chi^2^ test *p*-value = 0.014) but not active and non-active UC (Chi^2^ test *p*-value > 0.05) (Fig. 3B). Nevertheless, adjusted *p*-values did not highlight any significant differences for specific bifidobacterial species between non-active and active CD. The same bifidobacterial taxonomic profiles were identified in active CD and UC (Chi^2^ test *p*-value > 0.05) while non-active CD and UC significantly differed (Chi^2^ test *p*-value < 0.001) in *B. adolescentis* (adjusted *p*-value < 0.001) and *B. dentium* (adjusted *p*-value < 0.01). Indeed, *B. adolescentis* was predominant in non-active CD (40% of bifidobacteria isolated), whereas *B. dentium* was predominant in active CD (28%), and active (34%) and non-active UC (44%) (Fig. 3B).

### Age-related evolution in bifidobacteria profiles in IBD patients

**Fig. 4:**
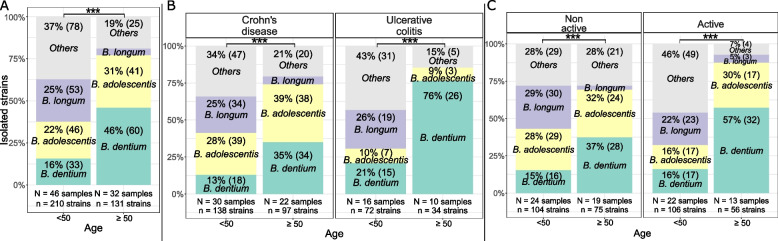
Characterization of the bifidobacteria profile in intestinal microbiota of IBD patients by age (**A**) for all patients, (**B**) per pathology and (**C**) per disease activity. Ratios are presented as relative proportion of bifidobacteria isolated (absolute number of bifidobacteria strains isolated). Ratios were not displayed for species with relative abundance ≤ 5%. N: number of samples from which strains were isolated. n: number of strains isolated. Chi.^2^ test *p*-values: *: 0.01–0.05; **: 0.001–0.01; ***: < 0.001; ns: > 0.05

In IBD patients, relative abundances of bifidobacteria species varied with age (Chi^2^ test *p*-value < 0.001 between patients < and ≥ 50 years old), notably with a significant increase in *B. dentium* in IBD patients above 50 years old, and a significant decrease in *B. longum* and *B.* *pseudocatenulatum* (adjusted *p*-values < 0.001 between groups < 50 and ≥ 50 years old) (Fig. 4A). Increase in *B. dentium* with age was observed in UC and CD (adjusted *p*-values < 0.001, Fig. 4B) and in non-active (adjusted *p*-values < 0.01) and active (adjusted *p*-values < 0.001) IBD patients (Fig. 4C). In the same manner, decrease in *B. longum* with age was observed in UC (adjusted *p*-values < 0.001) and CD (adjusted *p*-values < 0.01, Fig. 4B) and in non-active (adjusted *p*-values < 0.01) and active (adjusted *p*-values < 0.001) IBD patients (Fig. 4C). These observations did not seem to correlate with duration of the pathology. Mean age of patients was not significantly different per pathology groups (Wilcoxon test *p*-value = 0,3) and differences in microbiota composition with age did not seem to correlate with microbiota variations according to the duration of the pathology (data not shown).

## Functional characterization of bifidobacteria isolated from IBD patients

### In vitro growth capacities

**Fig. 5:**
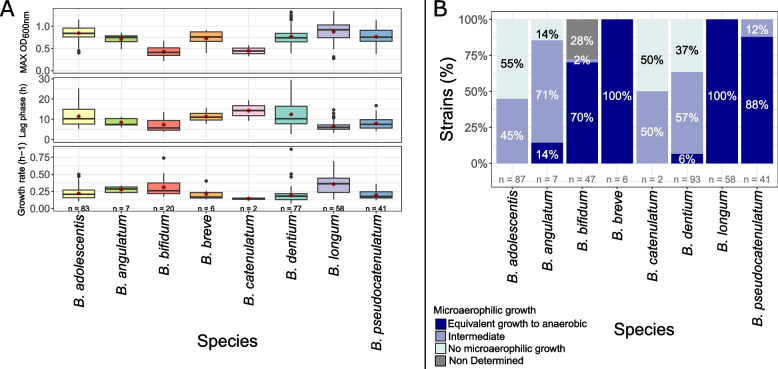
Growth characterization of bifidobacteria in (**A**) anaerobic and (**B**) microaerophilic (under 5% 0_2_) conditions. **A** Growth parameters were not determined for strains with Max OD_600nm_ < 0.2 or that did not reach end of exponential phase after 48h. Boxplots represent median (solid line), first and third quartile (lower and upper boundaries), lower and upper limits (whiskers, defined by 1.5* inter quartile range) and outliers (dotpoints). (B) Not determined: strains that did not present growth in control condition (anaerobic) to calculate microaerophilic growth ratio

As bifidobacteria were isolated from intestinal inflammatory environments, bacteria fitness was evaluated by determining anaerobic growth kinetics and oxygen tolerance to evidence possible differences according to isolation origin. In vitro growth parameters were evaluated by following growth kinetics in anaerobic and microaerophilic (5% O_2_) conditions. Growth abilities largely differed between strains, but trends were observed per species. Globally, in anaerobic conditions, *B. longum* presented the best growth abilities with reduced lag phases (average of 6.5h), highest growth rates (average of 0.36 h^−1^) and highest OD_600nm_ reached (average of OD_600nm_ = 0.9). For other species, lag phases were around 10h, growth rated around 0.25h^−1^ and maximum OD_600nm_ were heterogeneously distributed among strains, but generally reduced for *B. bifidum* strains (OD_600nm_ = 0.4 on average) (Fig. 5A).

In microaerophilic conditions (5% O_2_), 100% (*n* = 58 strains) of *B. longum*, 100% (*n* = 6) of *B. breve*, 88% (*n* = 36) of *B. pseudocatenulatum* and 70% (*n* = 33) of *B. bifidum* presented equivalent growth capacities to anaerobic conditions. *B. adolescentis* and *B. dentium* showed limited oxygen tolerance with respectively 55% (*n* = 48) of *B. adolescentis* strains and 37% (*n* = 34) of *B. dentium* strains exhibiting no growth in microaerophilic conditions (Fig. 5B).

Bacterial fitness in anaerobic conditions and oxygen tolerance did not depend on isolation origins: no significant differences were found between strains isolated from CD or UC patients in active or non-active disease.

### Exopolysaccharide synthesis

**Fig. 6:**
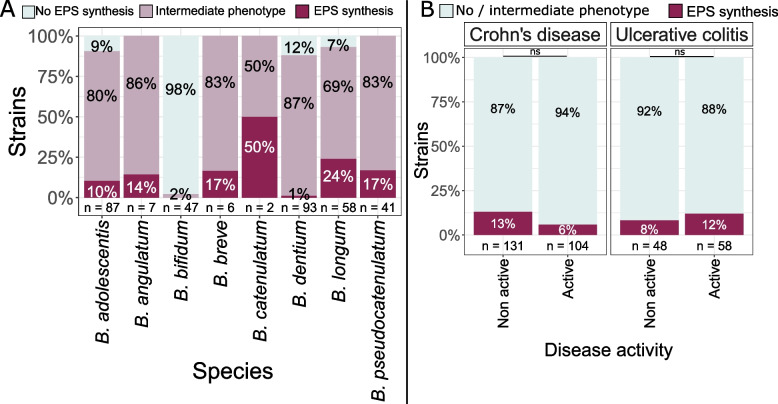
EPS production on 20g/L glucose MRS agar plates (**A**) per bifidobacterial species and (**B**) according to strain origin (pathology and disease activity). Chi.^2^ test *p*-values: *: 0.01–0.05; **: 0.001–0.01; ***: < 0.001; ns: > 0.05

EPS secretion was evaluated by observing the aspects of colonies on agar plates containing different carbohydrate sources. EPS synthesis was strain dependent with a wide variety of phenotypes within the same species. None of the *B. bifidum* (*n* = 47) and only one *B. dentium* (*n* = 93) tested presented an EPS producing phenotype on the 20g/L glucose MRS agar plates. *B. longum* strains had high EPS producing potential, with 24% (*n* = 14) of *B. longum* strains producing EPS. We also identified 9 *B. adolescentis*, 1 *B. angulatum*, 1 *B. breve*, 1 *B. catenulatum* and 7 *B. pseudocatenulatum* EPS producing strains (Fig. 6A). Similar results were obtained for EPS synthesis when growing on other carbohydrate sources i.e. lactose, maltose and sucrose (data not shown).

For 32 out of the 34 strains with an EPS producing phenotype, potential EPS encoding gene clusters were detected in silico. All genomic clusters contained a priming glycosyltransferase gene: *rfbP*, encoding an undecaprenyl-phosphate sugar phospho-transferase were mainly identified in *B. adolescentis*, *B.* *pseudocatenulatum* and *B.* *dentium* while *cpsD*, a galactosyl-transferase, was predominant in *B. longum*, *B. angulatum* and *B.* *catenulatum* (Supplementary Figure S1). The genetic regions surrounding the primary glycosyltransferase gene displayed different structural organizations, but contained characteristic genes involved in EPS synthesis (including glycosyltransferases, genes involved in rhamnose biosynthesis and transport related flippases or ABC transporters). In all 13 *B. longum* EPS gene clusters, several glycosyltransferases and chain length determinant proteins were identified. Flippases or ABC transporter encoding genes were found in 5 EPS gene clusters and genes described as involved in polysaccharide biosynthesis in 8 EPS gene clusters. *B.* *pseudocatenulatum* strains also presented potential complete EPS gene clusters as the 7 genetic regions identified all contained a priming glycosyltransferase, glycosyltransferases and other polysaccharide synthesis related genes. Five out of the 7 potential EPS gene clusters also presented a flippase encoding gene. In *B. adolescentis*, in the surrounding regions of a potential priming glycosyltransferase identified in 9 strains, only one presented other glycosyltransferases and a polysaccharide biosynthesis related protein. For the other potential EPS encoding regions, a chain-length determinant protein like gene was identified and in 6 regions, rhamnose biosynthesis genes were highlighted. Finally, potential complete EPS gene clusters were identified in 1 *B.* *angulatum*, 1 *B.* *catenulatum* and 1 *B.* *dentium* strain, containing a priming glycosyltransferase, several glycosyltransferases, polysaccharide biosynthesis protein and transporter encoding genes (Fig. S1).

Regarding the origin of the strains, EPS production frequency in bifidobacteria did not correlate with isolation origin. No differences were observed between strains isolated from active and non-active CD or from active and non-active UC (Chi^2^ test *p*-value > 0.1) (Fig. 6B). Correlations were neither found according to other isolation origin parameters (age, length of pathology, overall differences between pathologies or disease activity).

### Antibacterial activity

**Fig. 7:**
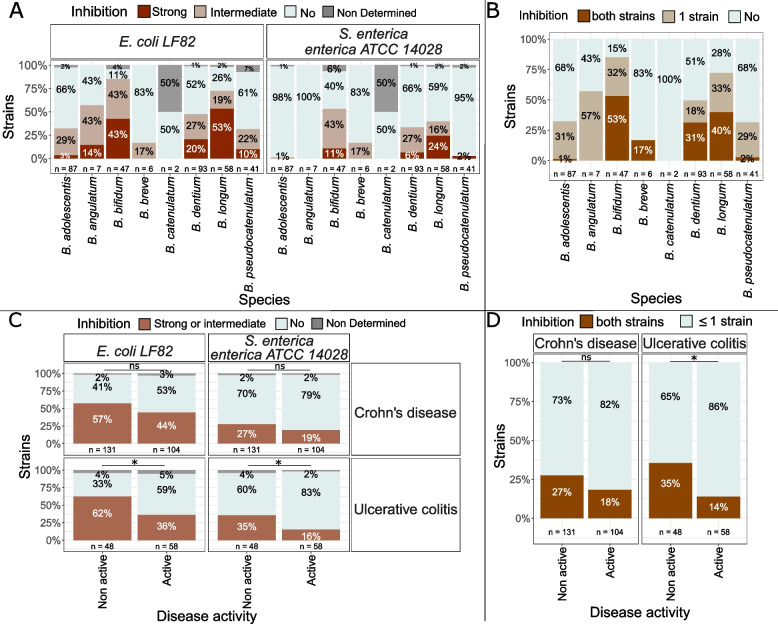
Inhibition of E. coli LF82 and Salmonella enterica ATCC 14028 by bifidobacteria. **A** Inhibition profiles by bifidobacteria per species and per pathogen tested. **B** Global inhibition profiles by bifidobacteria species (number of pathogens inhibited). **C** Inhibition profiles by bifidobacteria per pathogen tested according to isolation origin (pathology and disease activity). **D** Global inhibition profiles according to isolation origin (pathology and disease activity). Chi^2^ test *p*-values: *: 0.01–0.05; **: 0.001–0.01; ***: < 0.001; ns: > 0.05. n: number of strains. Not determined: insufficient growth of bifidobacterial strain in experiment conditions

Antibacterial activity of the 341 bifidobacteria was evaluated by co-culture and inhibition halo observation for *Salmonella enterica* subsp. *enterica* ATCC 14028 and *Escherichia coli* LF82 strains (Fig. 7A, B). Most strains with high anti-pathogenic activity against both strains were *B. bifidum* (53% of strains inhibited both pathogens), *B. longum* (40%) and *B. dentium* (31%) while *B. adolescentis*, *B. angulatum*, *B. breve*, *B.* *catenulatum* and *B. pseudocatenulatum* strains had very limited antibacterial activity, with none or only 1 strain per species able to inhibit both pathogens (Fig. 7A, B).

Bifidobacterial strains inhibiting both *E. coli* and *S. enterica* were enriched in non-active UC compared to active UC (Chi^2^ test *p*-value = 0.017). In UC, bifidobacteria inhibiting both *E. coli* (Chi^2^ test *p*-value = 0.025) and *S. enterica* (Chi^2^ test *p*-value = 0.037) were more prevalent in non-active disease compared to active disease. In CD, the same trend was observed but without statistical significance (Fig. 7 C, D). No correlations between anti-bacterial activity and other strain origin characteristics were identified.

**Fig. 8:**
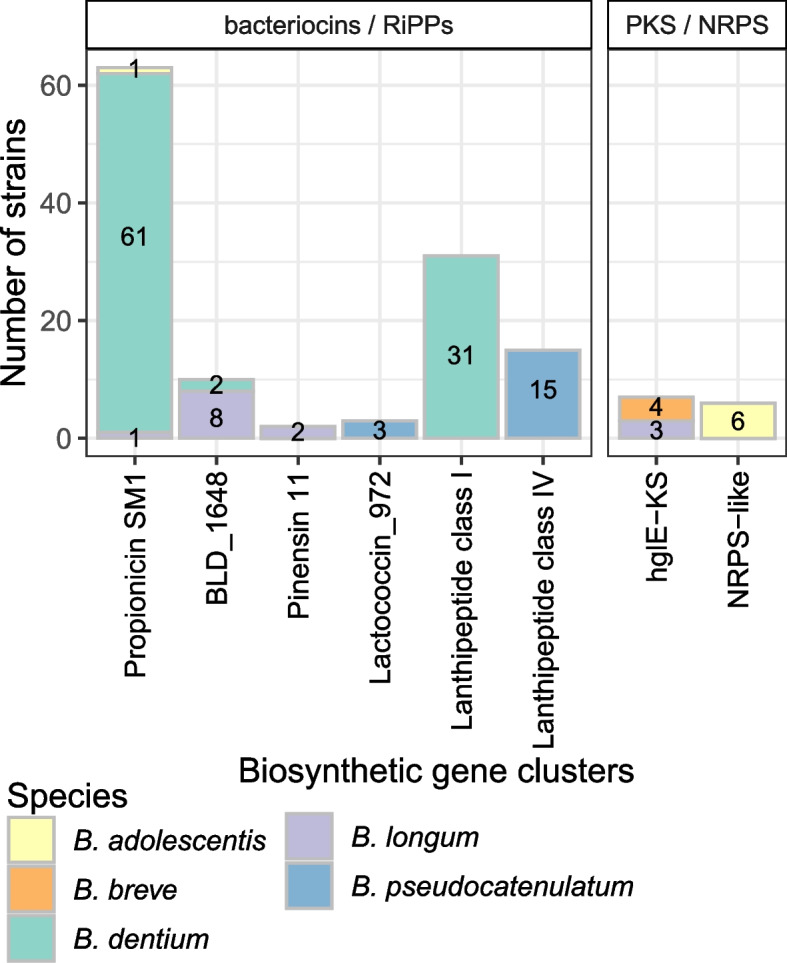
Biosynthetic gene clusters identified in bifidobacteria genomes by combined analysis with antiSMASH and BAGEL4

In addition to in vitro antimicrobial screening, biosynthetic gene clusters (BGCs) potentially involved in the synthesis of antimicrobial metabolites were searched by antiSMASH and BAGEL4 bioinformatic tools. BGCs were identified in 115 genomes, corresponding to 34% of all strains (Fig. 8). Gene clusters identified are represented in supplementary figures S2 and S3. Mainly, a propionicin SM1 like gene cluster was identified in 61 *B. dentium* genomes (66% of all *B. dentium* strains), 1 *B. longum* and 1 *B. adolescentis* genome. The lanthipeptide BLD_1648 gene cluster was detected in 8 *B. longum* and 2 *B. dentium* genomes. Other lanthipeptide gene clusters were identified in 31 *B. dentium* (class I lanthipeptides) and 15 *B.* *pseudocatenulatum* (class IV lanthipeptides). Furthermore, pinensin-like and lactoccoccin like gene clusters were found respectively in 2 *B. longum* and 3 *B. pseudocatenulatum* genomes. Finally, 6 Non-Ribosomal Peptide Synthetase (NRPS) gene clusters were identified in *B. adolescentis* genomes and 7 polyketide synthetases (PKS) in *B. breve* and *B. longum* strains.

Among the 25 bifidobacteria that presented strong inhibition against both *E. coli* and *S. enterica*, only 8 (6 *B. longum* and 2 *B. dentium*) presented BGCs. Among these strains, we find the 2 *B. longum* strains for which the pinensin 11 BGCs were identified and the 3 *B. longum* with the hglE-KS BGCs. Furthermore, one *B. longum* strain with high antimicrobial activity encoded the BLD_1648 gene cluster. The 2 *B. dentium* strains with high antimicrobial activity in vitro both encoded the propionicin SM1 gene cluster and one of them also presented an unknown class I lanthipeptide BGC. From the 56 strains inhibiting only one of the two pathogens in vitro, BGCs were identified in 15 strains, among which 11 the propionicin SM1 and 4 the BLD_1648 gene cluster. In contrast, among the 155 strains that did not have antimicrobial activity in vitro against *E. coli* or *S. enterica*, 63 strains encoded for BGCs (Propionicin SM1, BLD_1648, Lactoccocin_972, different lanthipeptides, KPS and NRPS gene clusters). Further analysis will be needed to evaluate if these strains indeed produce these antimicrobial compounds in vitro, their exact structure and spectrum of action.

### Immunomodulatory activity

**Fig. 9:**
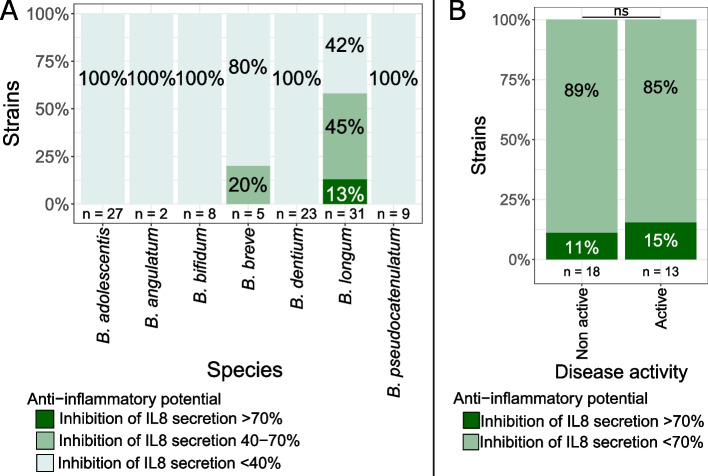
Immunomodulatory activity of bifidobacteria by inhibition of IL8 secretion of HT29 cells. (**A**) Immunomodulatory profile of all bifidobacteria per species. (**B**) Immunomodulatory profile of B. longum according to disease activity. Chi^2^ test *p*-values: *: 0.01–0.05; **: 0.001–0.01; ***: < 0.001; ns: > 0.05. n: number of strains

Immunomodulation was determined by assessing inhibition of LPS-induced IL-8 secretion by HT-29 human epithelial cells after treatment with a selection of 105 genetically different bifidobacteria. Herein, from the seven bifidobacterium species isolated, only *B. longum* and *B. breve* strains presented significant anti-inflammatory activity. Only 4 strains (all *B. longum*) out of the 105 strains tested presented high anti-inflammatory activity (> 70% inhibition of IL-8 secretion compared to LPS-induced control). *B. longum* thus presented the highest anti-inflammatory activity (13% of *B. longum* strains tested presented high anti-inflammatory activity). An additional 45% of the *B. longum* (*n* = 14) presented moderate anti-inflammatory activity (inhibition of 40 to 70% of IL-8 secretion) (Fig. 9A).

Regarding the origin of the strains, no differences in anti-inflammatory activity were found between *B. longum* isolated from patients in active er non active disease (Chi^2^ test *p*-value = 1, Fig. 9B) or between UC or CD (Chi^2^ test *p*-value = 0.7, data not shown). Statistical analyses were not performed according to pathology and disease activity combined due to reduced strain number per group.

## Discussion

**Fig. 10:**
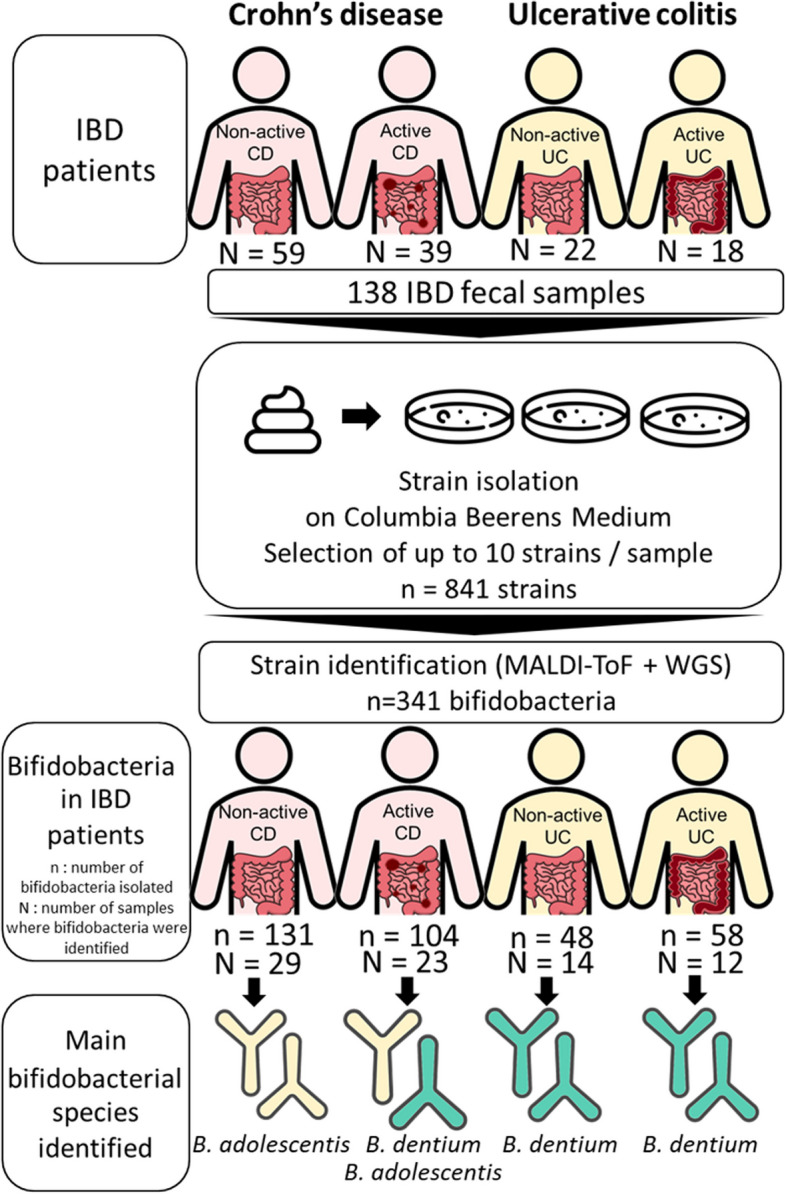
Summary of bifidobacteria characterization in IBD patients

To our knowledge, only few studies were published focusing specifically on taxonomic diversity of bifidobacteria in intestinal microbiota of IBD patients at species level. These studies included a limited number of patients (< 15 IBD patients), and do not distinguish active from non-active IBD [12, 13]. Here, we set up a culturomics approach to give insight into the taxonomic diversity of bifidobacteria in the IBD microbiota of a large group of patients (103 patients), in active (57 samples) or non-active IBD (81 samples) (Fig. 10). As fecal samples were collected as secondary use as part of a medical follow-up, the study design included only IBD patients and no healthy controls, setting the focus of this work to comparisons between active and non-active CD and UC. We collected a large metadata set allowing us to analyze bifidobacteria microbiota in IBD patients according to different parameters such as type of pathology, disease activity, disease location, age or duration of pathology. We defined disease activity considering fecal calprotectin quantification (a specific marker of gastrointestinal inflammation) and symptomatic observations. Calprotectin levels are correlated with endoscopic activity but present limitations in terms of specificity and individual variation. Fecal calprotectin levels described in the literature to predict disease activity, mucosal healing or disease relapse risk go from 50 µg/g to 500 µg/g, with sensitivities going from 61 to 100% and specificities between 43 and 90% [59–61]. In our analysis, we defined non-active UC or CD as patients with fecal calprotectin levels strictly lower than 250 µg/g that did not experience any gastrointestinal disorders. Active CD or UC was defined as patients with fecal calprotectin levels equal to or higher than 250 µg/g (i.e. patients with inflammation in the gastro-intestinal tract, with or without gastrointestinal disorders).

With this culturomics approach, bacterial enumeration on bifidobacteria enrichment medium and total anaerobic flora were similar between patients in CD and UC, regardless of disease activity. This was consistent with the finding of Premkumar et al*.*, who did not identify differences in *Bifidobacterium* abundance between healthy subjects, CD and UC patients by qPCR [62]. Nevertheless, after taxonomic identification of isolated strains, only 41% of isolated strains appeared to be bifidobacteria and for 44% of the samples, no bifidobacteria were isolated. Isolates were randomly selected from Columbia Beerens agar plates, but as this medium was not fully selective for bifidobacteria, some minor bifidobacteria populations in fecal samples might not have been evidenced here, especially in the case of a high abundance of lactic acid bacteria with favored growth on Columbia Beerens Medium. This created a bias in our study due to the culturomics approach chosen, in which only part of all cultivable bifidobacteria present might have been identified.

Differences in bifidobacteria microbiota composition according to pathology and IBD activity in this study were observed mainly between the two major species isolated, *B. adolescentis* and *B. dentium*, 2 closely related species belonging to the *B. adolescentis* phylogenetic group [63]. *B. dentium* were enriched in UC, both in non-active and active disease. In contrast to our results, studies of Gueimonde et al. and Duranti et al., analyzing bifidobacteria profiles in IBD patients, did not identify *B. dentium* strains [12, 13]. We also highlighted increased *B. dentium* relative abundances in patients above 50 years old. These results are consistent with studies on healthy subjects showing increased *B. dentium* in elderly [64, 65]. *B. dentium* are known as members of the human intestinal microbiota but are not generally found as the major bifidobacteria species present. Furthermore, *B. dentium* was first identified in dental caries and several studies confirmed their cariogenic properties (ability to survive in the human oral cavity and acidogenic potential) [66, 67]. Associations were established between IBD and oral diseases (periodontitis, higher caries incidence) [68–70], which could correlate with the high *B. dentium* ratios found in IBD patients in our study. Nevertheless, *B. dentium* are also part of the human healthy microbiota and have shown beneficial effects in animal models of IBD. In vitro, *B. dentium* strains have been shown to positively influence the epithelial barrier function by decreasing gut permeability [71–74], and regulating mucin synthesis [75]. In vivo, strains of *B. dentium* were able to relieve colitis symptoms in a DSS stimulated mice model [72], decreased weight loss and levels of TNF, IL-8 and IL-6 inflammatory markers in a TNBS-induced colitis mice model [76]. These data suggest that higher abundance of *B. dentium* in IBD patients should not only be considered as a marker of dysbiosis but could play a beneficial role.

Furthermore, this study found increased *B. adolescentis* relative abundances in CD compared to UC, especially in non-active CD. This observation was not consistent with studies of Gueimonde et al. and Duranti et al., who did not reveal *B. adolescentis* as specific markers of IBD [12, 13] while Barberio et al*.* showed increased *B. adolescentis* in non-active UC compared to active UC and healthy controls [77]. *B. adolescentis* is generally known as one of the main bifidobacterial species of the human gut microbiota and largely studied for its potential probiotic applications. One of the most evidenced functionalities of *B. adolescentis* is γ-aminobutyric acid secretion, a major inhibitory neurotransmitter that could be responsible for anxiolytic and antidepressant effects. Furthermore, *B. adolescentis* were shown to secrete vitamin B9 and have beneficial effects on the gut barrier in in vitro colitis models by increasing mucus layer and present immunoregulatory effects (stimulation of regulatory T cells, reduction of nuclear factor κB) (reviewed in [78]). These beneficial characteristics of *B. adolescentis* might explain their prevalence in non-active CD.

The higher *B. dentium* and *B. adolescentis* abundances identified in our analysis could be specific to our population, with environmental and geographic influences, or depend on the isolation method used. All bifidobacteria species presented sufficient growth on the selective medium used (Columbia Beerens medium) but no in-depth analysis was performed on the selectivity of this medium. However, our culturomics approach did not seem to favor oxygen tolerant species,*B. dentium* and *B. adolescentis*, as these species are among the most oxygen sensitive bifidobacteria in our in vitro functional characterization. Comparison of different studies describing the taxonomic diversity of bifidobacteria in IBD remains challenging as other studies are mainly based on metagenomic approaches andalso include many biases during sample treatment and bioinformatic analysis [21, 22]. This complicates identification and semi-quantitative analysis for minor populations of the intestinal microbiota.

During functional characterization of our bifidobacteria collection, we confirmed previously known phenotypes and functions of bifidobacteria (oxygen tolerance, exopolysaccharide synthesis, antimicrobial activity, and immunomodulatory effect) on a large number of strains.

Exopolysaccharide production in vitro was associated with potential EPS gene clusters in different *B. longum*, *B. pseudocatenulatum* and *B. adolescentis* strains. *B. longum* and *B. pseudocatenulatum* presented EPS gene clusters similar to those already described in the literature [23, 44, 45], containing genes encoding for priming glycosyltransferases, glycosyltransferases, rhamnose biosynthesis, chain length determinant proteins and other polysaccharide biosynthesis proteins. Nevertheless, no glycosyltransferase encoding genes were identified in *B. adolescentis* potential EPS gene clusters. Further in vitro and in silico research will be needed to correlate EPS producing phenotypes to gene clusters and identify if the genetic regions identified here are related to EPS synthesis in *B. adolescentis* strains. Besides highlighting the antimicrobial activity of *B. longum* and *B. bifidum*, already described in literature, we evidenced probiotic potential of *B. dentium* strains through inhibition of *E. coli* LF82 and *S. enterica ATCC* 14028. Antimicrobial activity of the bifidobacterial strains can be associated with several mechanisms such as the production of acetic and lactic acids, but also synthesis of specific antimicrobial compounds. 115 bifidobacterial genomes presented BGCs encoding potential bacteriocins, ribosomally synthesized and post-translationally modified peptides or non ribosomally synthesized peptides (NRPS, PKS). Most noticeable, 66% of the *B. dentium* strains could potentially produce a propionicin SM1 like bacteriocin.

Most of the probiotic characteristics assessed were already largely described in literature, but herein, this study evaluated if these characteristics were associated with the isolates’ origin (type of patient, disease activity, age, pathology duration). No differences were observed in bacterial fitness, oxygen tolerance, EPS synthesis and immunomodulatory activity between isolation origin. Bifidobacteria isolated from non-active UC presented increased anti-pathogenic activity against both Adherent Invasive *E. coli* LF82 and *S. enterica* ATCC 14028 compared to bifidobacteria isolated from active UC. More generally, our study highlighted that bifidobacteria, even when isolated from inflamed gastrointestinal environments from active IBD patients present potential probiotic characteristics. *B. longum* presented the highest ratios of strains for EPS synthesis, antibacterial activity, and anti-inflammatory capacities. The potential probiotic *B. longum* strains were isolated from both active and non-active IBD patients. This supports the idea that strains with potential probiotic capacities can be isolated even from patients in active disease state. Even if these strains did not manage IBD symptoms when naturally occurring in the IBD intestinal microbiota, they could have beneficial effects on gut health when supplemented in higher amount or in other patients, where other interactions could occur with the host microbiota and immune system. Further pre-clinical and clinical studies could confirm the potential use of IBD isolated *B. longum* strains for applications as probiotics in general gut health and/or in IBD treatment.

## Conclusions

We conducted a culturomics approach to characterize the diversity of bifidobacteria and their functionality in the microbiota of active and non-active IBD patients. Relative bifidobacteria abundances were high in *B. dentium* and *B. adolescentis*. *B. dentium* were mainly associated to UC and *B. adolescentis* to CD whereas no specific bifidobacterial taxonomic profiles were highlighted according to active or non-active disease states. Additionally, *B. dentium* relative abundances increased with age, along with a decrease in *B. longum* and *B. pseudocatenulatum*.. Correlations were found between the anti-pathogenic capacities of the strains and their origin, but overall, we highlighted that bifidobacteria with possible probiotic potential (antipathogenic activity, EPS production and anti-inflammatory activity) were isolated from both non-active and active CD and UC patients. We identified interesting *B. longum* strains for further characterization and potential application as probiotics for human gut health.

### Supplementary Information

Suplementary material 1.

Suplementary material 2.

## Data Availability

Data supporting the findings of this study are available within the supplementary file provided. Associated DNA sequences were deposited in SRA under accession number PRJNA1090283.

## Abbreviations

BGC: Biosynthetic Gene Cluster
CD: Crohn’s disease
EPS: Exopolysaccharides
IBD: Inflammatory Bowel Diseases
LPS: Lipopolysaccharide
MOI: Multiplicity Of Infection
MRS: De Man Ragosa and Sharpe Medium
NRPS: Non Ribosomal Peptide Synthetase
OD_600nm_: Optical Density at 600 nm
OD_620nm_: Optical Density at 620 nm
PKS: Polyketide Synthase
UC: Ulcerative colitis

## References

[1] Neurath MF, Vieth M (2023). Different levels of healing in inflammatory bowel diseases: mucosal, histological, transmural, barrier and complete healing. Gut.

[2] Ananthakrishnan AN, Xavier RJ, Podolsky DK, Wang TC, Camilleri M, Lebwohl B,  Lok AS, Sandborn WJ, Wang KK, Wu  GD (2022). Inflammatory bowel diseases: pathogenesis. In. Yamada’s Textbook of Gastroenterology, Seventh Edition.

[3] Bruner LP, White AM, Proksell S (2023). Inflammatory bowel disease. Prim Care.

[4] Flynn S, Eisenstein S (2019). Inflammatory bowel disease presentation and diagnosis. Surg Clin North Am.

[5] Nishida A, Inoue R, Inatomi O, Bamba S, Naito Y, Andoh A (2018). Gut microbiota in the pathogenesis of inflammatory bowel disease. Clin J Gastroenterol.

[6] Desreumaux P, Foligné B, Titecat M. Microbiote intestinal et santé humaine - Partie 2 Microbiote intestinal et pathologies - Chapitre - Maladies inflammatoires chroniques intestinales. Paris: Elsevier Masson; 2021.

[7] Lloyd-Price J, Arze C, Ananthakrishnan AN, Schirmer M, Avila-Pacheco J, Poon TW (2019). Multi-omics of the gut microbial ecosystem in inflammatory bowel diseases. Nature.

[8] Aldars-García L, Chaparro M, Gisbert JP (2021). Systematic review: the gut microbiome and its potential clinical application in inflammatory bowel disease. Microorganisms.

[9] Vestergaard MV, Allin KH, Eriksen C, Zakerska-Banaszak O, Arasaradnam RP, Alam MT (2023). Gut microbiota signatures in inflammatory bowel disease. United European Gastroenterol J.

[10] Banzragch M, Sanli K, Stensvold CR, Kurt O, Ari S (2023). Metabarcoding of colonic cleansing fluid reveals unique bacterial members of mucosal microbiota associated with inflammatory bowel disease. Scand J Gastroenterol.

[11] Wang W, Chen L, Zhou R, Wang X, Song L, Huang S (2014). Increased proportions of *Bifidobacterium* and the *Lactobacillus* group and loss of butyrate-producing bacteria in inflammatory bowel disease. J Clin Microbiol.

[12] Gueimonde M, Ouwehand A, Huhtinen H, Salminen E, Salminen S (2007). Qualitative and quantitative analyses of the bifidobacterial microbiota in the colonic mucosa of patients with colorectal cancer, diverticulitis and inflammatory bowel disease. World J Gastroenterol.

[13] Duranti S, Gaiani F, Mancabelli L, Milani C, Grandi A, Bolchi A (2016). Elucidating the gut microbiome of ulcerative colitis: bifidobacteria as novel microbial biomarkers. FEMS Microbiol Ecol.

[14] Jakubczyk D, Leszczyńska K, Górska S (2020). The effectiveness of probiotics in the treatment of inflammatory bowel disease (IBD)-a critical review. Nutrients.

[15] Yao S, Zhao Z, Wang W, Liu X (2021). *Bifidobacterium longum*: protection against inflammatory bowel disease. J Immunol Res.

[16] Furrie E, Macfarlane S, Kennedy A, Cummings JH, Walsh SV, O’neil DA, Macfarlane GT. Synbiotic therapy (*Bifidobacterium longum*/Synergy 1) initiates resolution of inflammation in patients with active ulcerative colitis: a randomised controlled pilot trial. Gut. 2005. 10.1136/gut.2004.044834. 10.1136/gut.2004.044834 PMC1774839 15647189

[17] Ishikawa H, Matsumoto S, Ohashi Y, Imaoka A, Setoyama H, Umesaki Y (2011). Beneficial effects of probiotic bifidobacterium and galacto-oligosaccharide in patients with ulcerative colitis: a randomized controlled study. Digestion.

[18] Groeger D, O’Mahony L, Murphy EF, Bourke JF, Dinan TG, Kiely B (2013). *Bifidobacterium infantis* 35624 modulates host inflammatory processes beyond the gut. Gut Microbes.

[19] Tamaki H, Nakase H, Inoue S, Kawanami C, Itani T, Ohana M (2016). Efficacy of probiotic treatment with *Bifidobacterium longum* 536 for induction of remission in active ulcerative colitis: A randomized, double-blinded, placebo-controlled multicenter trial. Dig Endosc.

[20] Steed H, Macfarlane GT, Blackett KL, Bahrami B, Reynolds N, Walsh SV (2010). Clinical trial: the microbiological and immunological effects of synbiotic consumption - a randomized double-blind placebo-controlled study in active Crohn’s disease. Aliment Pharmacol Ther.

[21] Bharti R, Grimm DG (2021). Current challenges and best-practice protocols for microbiome analysis. Brief Bioinform.

[22] Roume H, Mondot S, Saliou A, Le Fresne-Languille S, Doré J (2023). Multicenter evaluation of gut microbiome profiling by next-generation sequencing reveals major biases in partial-length metabarcoding approach. Sci Rep.

[23] Hidalgo-Cantabrana C, Sánchez B, Milani C, Ventura M, Margolles A, Ruas-Madiedo P (2014). Genomic overview and biological functions of exopolysaccharide biosynthesis in *Bifidobacterium* spp. Appl Environ Microbiol.

[24] Pyclik M, Srutkova D, Schwarzer M, Górska S (2020). Bifidobacteria cell wall-derived exo-polysaccharides, lipoteichoic acids, peptidoglycans, polar lipids and proteins – their chemical structure and biological attributes. Int J Biol Macromol.

[25] Lee JG, Han DS, Jo SV, Lee AR, Park CH, Eun CS, Lee Y (2019). Characteristics and pathogenic role of adherent-invasive *Escherichia coli* in inflammatory bowel disease: Potential impact on clinical outcomes. PLoS One.

[26] López-Siles M, Camprubí-Font C, Del Pulgar G, Eva M, Sabat Mir M, Busquets D, Sanz Y, Prevalence M-M (2022). Abundance, and virulence of adherent-invasive escherichia coli in ulcerative colitis, colorectal cancer, and coeliac disease. Front Immunol.

[27] Martinez-Medina M, Aldeguer X, Lopez-Siles M, González-Huix F, López-Oliu C, Dahbi G (2009). Molecular diversity of Escherichia coli in the human gut: new ecological evidence supporting the role of adherent-invasive E. coli (AIEC) in Crohn’s disease. Inflamm Bowel Dis.

[28] Callaway TR, Edrington TS, Anderson RC, Byrd JA, Nisbet DJ (2008). Gastrointestinal microbial ecology and the safety of our food supply as related to *Salmonella*. J Anim Sci.

[29] Coburn B, Grassl GA, Finlay BB (2007). *Salmonella*, the host and disease: a brief review. Immunol Cell Biol.

[30] Park SW, Choi YH, Gho JY, Kang GA, Kang S-S (2023). Synergistic inhibitory effect of *Lactobacillus* cell lysates and butyrate on poly I:C-induced IL-8 production in human intestinal epithelial cells. Probiotics Antimicrob Proteins.

[31] Anwar M, Mros S, McConnell M, Bekhit AEDA (2022). Effects of Taro (Colocasia esculenta) water-soluble non-starch polysaccharide, lactobacillus acidophilus, bifidobacterium breve, bifidobacterium infantis, and their synbiotic mixtures on pro-inflammatory cytokine interleukin-8 production. Nutrients.

[32] do Carmo FLR, Rabah H, Cordeiro BF, Da Silva SH, Pessoa RM, Fernandes SOA, et al. Probiotic Propionibacterium freudenreichii requires SlpB protein to mitigate mucositis induced by chemotherapy. Oncotarget. 2019. 10.18632/oncotarget.27319. 10.18632/oncotarget.27319 PMC6944450 31921383

[33] Tuo Y, Song X, Song Y, Liu W, Tang Y, Gao Y (2018). Screening probiotics from *Lactobacillus* strains according to their abilities to inhibit pathogen adhesion and induction of pro-inflammatory cytokine IL-8. J Dairy Sci.

[34] Beck PL, Cotton JA, Platnich JM, Muruve DA, Buret AG, Jijon H (2016). Interleukin-8 in gastrointestinal inflammation and malignancy: induction and clinical consequences. IJICMR.

[35] Okada T, Kanda T, Ueda N, Ikebuchi Y, Hashiguchi K, Nakao K, Isomoto H (2020). IL-8 and LYPD8 expression levels are associated with the inflammatory response in the colon of patients with ulcerative colitis. Biomed Rep.

[36] Roselli M, Finamore A, Britti MS, Mengheri E (2006). Probiotic bacteria *Bifidobacterium animalis* MB5 and *Lactobacillus rhamnosus* GG protect intestinal Caco-2 cells from the inflammation-associated response induced by enterotoxigenic Escherichia coli K88. Br J Ntr.

[37] Gewirtz AT, Rao AS, Simon PO, Merlin D, Carnes D, Madara JL, Neish AS (2000). Salmonella typhimurium induces epithelial IL-8 expression via Ca(2+)-mediated activation of the NF-kappaB pathway. J Clin Invest.

[38] Beerens H (1991). Detection of bifidobacteria by using propionic acid as a selective agent. Appl Environ Microbiol.

[39] Alatoom AA, Cunningham SA, Ihde SM, Mandrekar J, Patel R (2011). Comparison of direct colony method versus extraction method for identification of gram-positive cocci by use of Bruker Biotyper matrix-assisted laser desorption ionization-time of flight mass spectrometry. J Clin Microbiol.

[40] Jain C, Rodriguez-R LM, Phillippy AM, Konstantinidis KT, Aluru S (2018). High throughput ANI analysis of 90K prokaryotic genomes reveals clear species boundaries. Nat Commun.

[41] Sprouffske K, Wagner A (2016). Growthcurver: an R package for obtaining interpretable metrics from microbial growth curves. BMC Bioinformatics.

[42] Ruas-Madiedo P (2021). Detection, isolation, and purification of bifidobacterial exopolysaccharides. Methods Mol Biol.

[43] Seemann T (2014). Prokka: rapid prokaryotic genome annotation. Bioinformatics.

[44] Altmann F, Kosma P, O’Callaghan A, Leahy S, Bottacini F, Molloy E (2016). Genome analysis and characterisation of the exopolysaccharide produced by Bifidobacterium longum subsp. longum 35624™. PLoS One.

[45] Tahoun A, Masutani H, El-Sharkawy H, Gillespie T, Honda RP, Kuwata K (2017). Capsular polysaccharide inhibits adhesion of *Bifidobacterium longum* 105-A to enterocyte-like Caco-2 cells and phagocytosis by macrophages. Gut pathogens.

[46] Paysan-Lafosse T, Blum M, Chuguransky S, Grego T, Pinto BL, Salazar GA (2023). InterPro in 2022. Nucleic Acids Res.

[47] O’Riordan K, Fitzgerald GF (1998). Evaluation of bifidobacteria for the production of antimicrobial compounds and assessment of performance in cottage cheese at refrigeration temperature. J Appl Microbiol.

[48] van Heel AJ, de Jong A, Song C, Viel JH, Kok J, Kuipers OP (2018). BAGEL4: a user-friendly web server to thoroughly mine RiPPs and bacteriocins. Nucleic Acids Res.

[49] Blin K, Shaw S, Kloosterman AM, Charlop-Powers Z, van Wezel GP, Medema MH, Weber T (2021). antiSMASH 6.0: improving cluster detection and comparison capabilities. Nucleic Acids Res.

[50] Li S-C, Hsu W-F, Chang J-S, Shih C-K (2019). Combination of lactobacillus acidophilus and bifidobacterium animalis subsp. lactis shows a stronger anti-inflammatory effect than individual strains in HT-29 Cells. Nutrients.

[51] Arboleya S, Watkins C, Stanton C, Ross RP (2016). Gut bifidobacteria populations in human health and aging. Front Microbiol.

[52] Boesten RJ, Schuren FHJ, Willemsen LEM, Vriesema A, Knol J, de Vos WM (2011). *Bifidobacterium breve* - HT-29 cell line interaction: modulation of TNF-α induced gene expression. Benef Microbes.

[53] Bahrami B, Macfarlane S, Macfarlane GT (2011). Induction of cytokine formation by human intestinal bacteria in gut epithelial cell lines. J Appl Microbiol.

[54] Stöber H, Maier E, Schmidt H (2010). Protective effects of Lactobacilli, Bifidobacteria and Staphylococci on the infection of cultured HT29 cells with different enterohemorrhagic *Escherichia coli* serotypes are strain-specific. Int J Food Microbiol.

[55] Grimoud J, Durand H, de Souza S, Monsan P, Ouarne F, Theodorou V, Rogues C (2010). *In vitro* screening of probiotics and synbiotics according to anti-inflammatory and anti-proliferative effects. Int J Food Microbiol.

[56] Candela M, Perna F, Carnevali P, Vitali B, Ciati R, Gionchetti P (2008). Interaction of probiotic *Lactobacillus* and *Bifidobacterium* strains with human intestinal epithelial cells: adhesion properties, competition against enteropathogens and modulation of IL-8 production. Int J Food Microbiol.

[57] Riedel C-U, Foata F, Philippe D, Adolfsson O, Eikmanns B-J, Blum S (2006). Anti-inflammatory effects of bifidobacteria by inhibition of LPS-induced NF-kappaB activation. World J Gastroenterol.

[58] Aghamohammad S, Sepehr A, Miri ST, Najafi S, Pourshafie MR, Rohani M (2022). The potential role of Bifidobacterium spp. as a preventive and therapeutic agent in controlling inflammation via affecting inflammatory signalling pathways. Lett Appl Microbiol.

[59] Khaki-Khatibi F, Qujeq D, Kashifard M, Moein S, Maniati M, Vaghari-Tabari M (2020). Calprotectin in inflammatory bowel disease. Clin Chim Acta.

[60] Bertani L, Mumolo MG, Tapete G, Albano E, Baiano Svizzero G, Zanzi F (2020). Fecal calprotectin: current and future perspectives for inflammatory bowel disease treatment. Eur J Gastroenterol Hepatol.

[61] Mumolo MG, Bertani L, Ceccarelli L, Laino G, Di Fluri G, Albano E (2018). From bench to bedside: Fecal calprotectin in inflammatory bowel diseases clinical setting. World J Gastroenterol.

[62] Premkumar K, Ramadass B, Ramakrishna B (2022). Fecal microbiota in inflammatory bowel disease: Studies of lactobacillus and bifidobacteria diversity. Gastroenterol Hepatol Endosc Pract.

[63] Lugli GA, Milani C, Duranti S, Mancabelli L, Mangifesta M, Turroni F (2018). Tracking the taxonomy of the genus *Bifidobacterium* based on a phylogenomic approach. Appl Environ Microbiol.

[64] Kato K, Odamaki T, Mitsuyama E, Sugahara H, Xiao J, Osawa R (2017). Age-related changes in the composition of gut *Bifidobacterium* Species. Curr Microbiol.

[65] Yang B, Yan S, Chen Y, Ross RP, Stanton C, Zhao J (2020). Diversity of gut microbiota and bifidobacterial community of chinese subjects of different ages and from different regions. Microorganisms.

[66] Manome A, Abiko Y, Kawashima J, Washio J, Fukumoto S, Takahashi N (2019). Acidogenic potential of oral bifidobacterium and its high fluoride tolerance. Front Microbiol.

[67] de Matos BM, Brighenti FL, Do Thuy, Beighton D, Koga-Ito CY (2017). Acidogenicity of dual-species biofilms of bifidobacteria and Streptococcus mutans. Clin Oral Investig.

[68] Nijakowski K, Gruszczyński D, Surdacka A (2021). Oral health status in patients with inflammatory bowel diseases: a systematic review. Int J Environ Res Public Health.

[69] Lorenzo-Pouso AI, Castelo-Baz P, Rodriguez-Zorrilla S, Pérez-Sayáns M, Vega P (2021). Association between periodontal disease and inflammatory bowel disease: a systematic review and meta-analysis. Acta Odontol Scand.

[70] Tan CXW, Brand HS, Kalender B, de Boer NKH, Forouzanfar T, de Visscher JGAM (2021). Dental and periodontal disease in patients with inflammatory bowel disease. Clin Oral Investig.

[71] Pan M, Barua N, Ip M (2022). Mucin-degrading gut commensals isolated from healthy faecal donor suppress intestinal epithelial inflammation and regulate tight junction barrier function. Front Immunol.

[72] Zhao L, Xie Q, Etareri Evivie S, Liu D, Dong J, Ping L (2021). *Bifidobacterium dentium* N8 with potential probiotic characteristics prevents LPS-induced intestinal barrier injury by alleviating the inflammatory response and regulating the tight junction in Caco-2 cell monolayers. Food Funct.

[73] Engevik MA, Danhof HA, Hall A, Engevik KA, Horvath TD, Haidacher SJ (2021). The metabolic profile of *Bifidobacterium dentium* reflects its status as a human gut commensal. BMC Microbiol.

[74] Lo Conte M, Cosorich I, Ferrarese R, Antonini Cencicchio M, Nobili A, Palmieri V (2023). Alterations of the intestinal mucus layer correlate with dysbiosis and immune dysregulation in human type 1 diabetes. EBioMedicine.

[75] Engevik MA, Luk B, Chang-Graham AL, Hall A, Herrmann B, Ruan W, et al. *Bifidobacterium dentium* Fortifies the Intestinal Mucus Layer via Autophagy and Calcium Signaling Pathways. mBio. 2019. 10.1128/mBio.01087-19. 10.1128/mBio.01087-19 PMC6581858 31213556

[76] Engevik MA, Herrmann B, Ruan W, Engevik AC, Engevik KA, Ihekweazu F (2021). *Bifidobacterium dentium*-derived y-glutamylcysteine suppresses ER-mediated goblet cell stress and reduces TNBS-driven colonic inflammation. Gut Microbes.

[77] Barberio B, Facchin S, Patuzzi I, Ford AC, Massimi D, Valle G (2022). A specific microbiota signature is associated to various degrees of ulcerative colitis as assessed by a machine learning approach. Gut Microbes.

[78] Leser T, Baker A (2023). *Bifidobacterium adolescentis* – a beneficial microbe. Benef Microbes.

