# Supplementary material for: Taxonomic and phenotypic analysis of bifidobacteria isolated from IBD patients as potential probiotic strains
Source: BMC Microbiol. 2024 Jun 29;24:233. doi: 10.1186/s12866-024-03368-4 (PMC11218132; doi:10.1186/s12866-024-03368-4)
Supplement: Supplementary file 1 — Suplementary material 1. [file 12866_2024_3368_MOESM1_ESM.docx]

# Supplementary figures


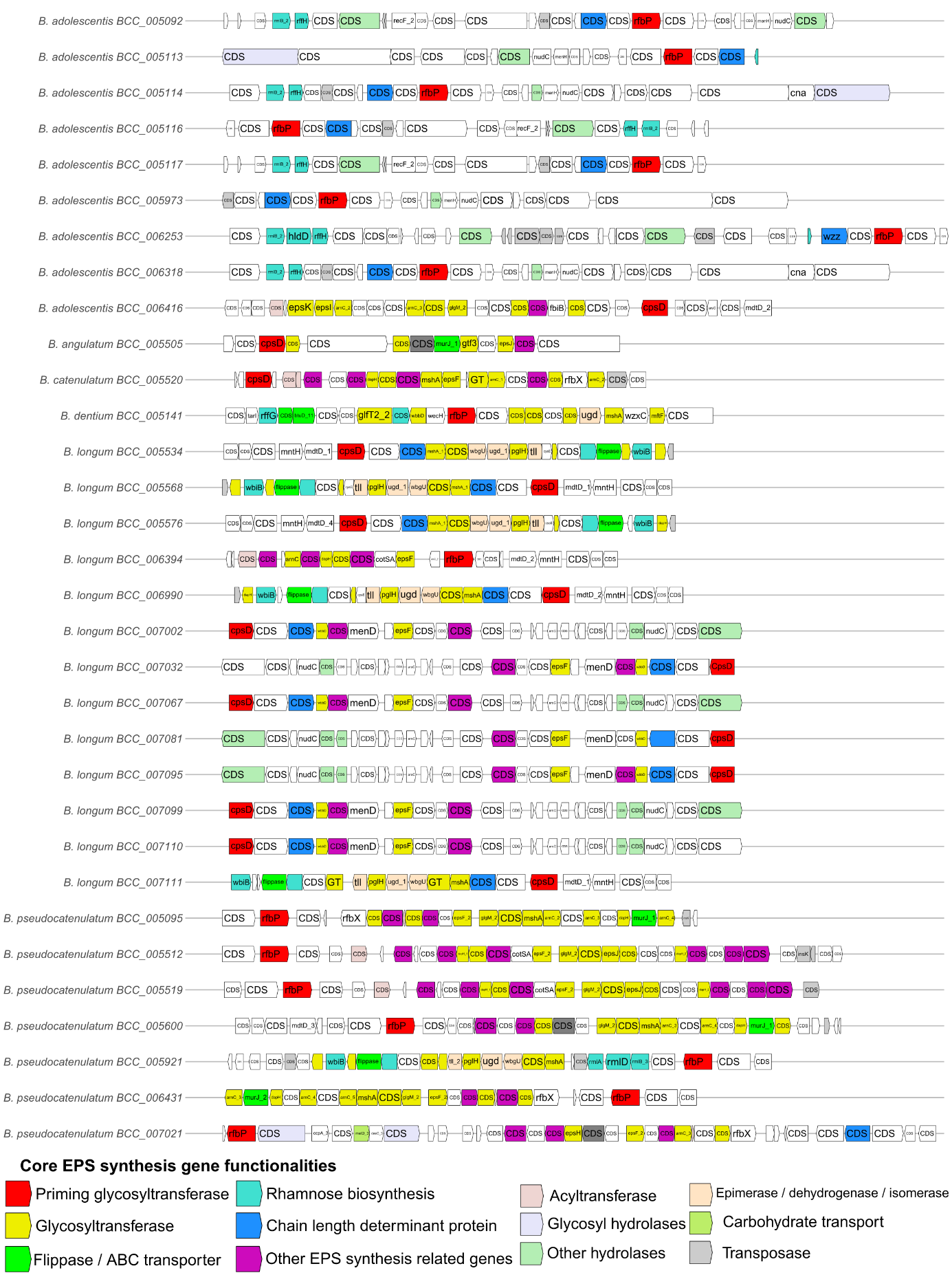


Fig. S1: Potential EPS gene clusters identified in EPS producing strains.


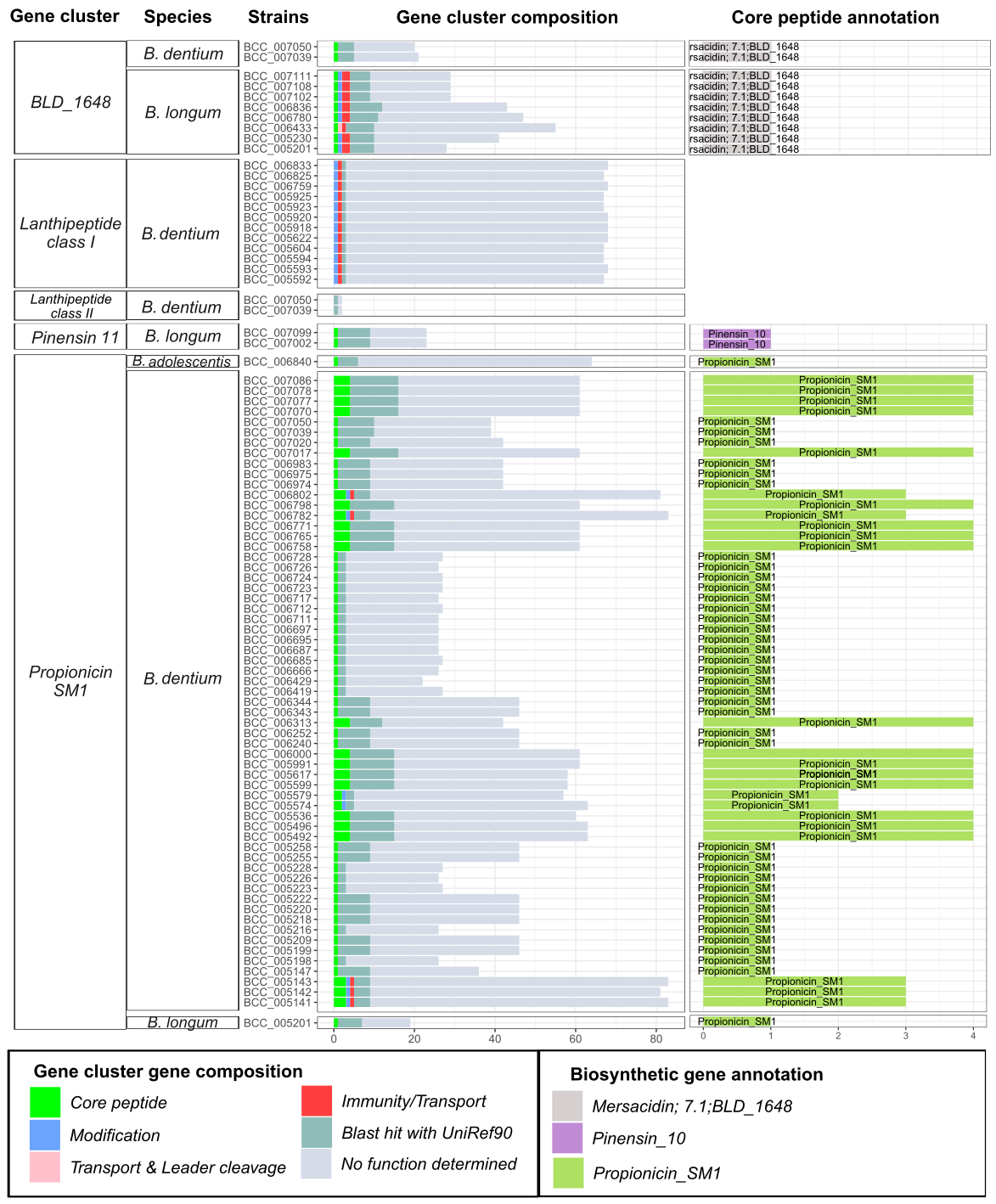


Figure S2: Gene clusters and core peptides identified in bifidobacterial genomes by BAGEL4 analysis.


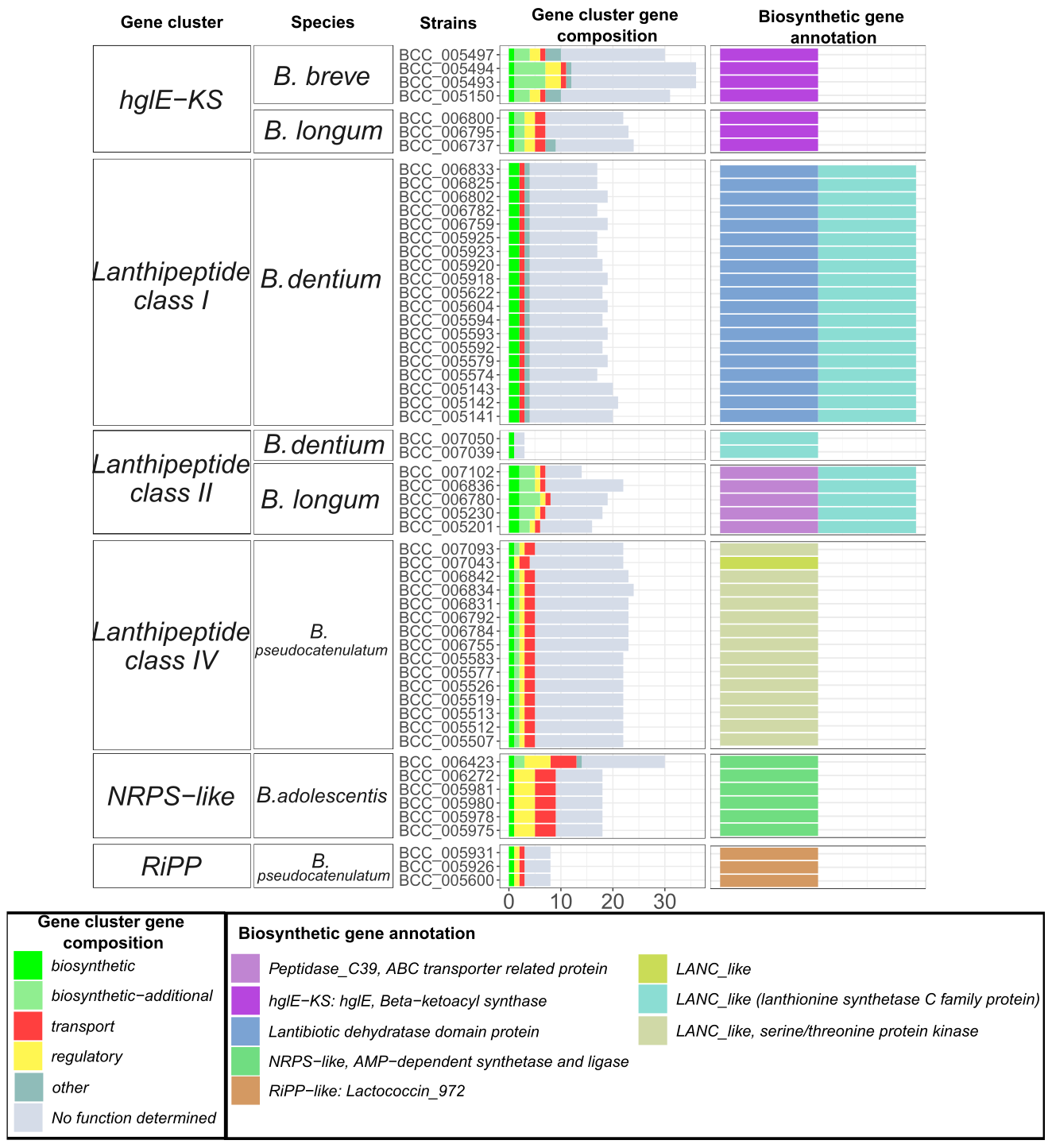


Figure S3: Biosynthetic gene clusters identified in bifidobacterial genomes by antiSMASH analysis.
